# Editorial: The interaction between self and other in the clinical setting: the role of inter-subjectivity, volume I

**DOI:** 10.3389/fpsyg.2026.1920628

**Published:** 2026-09-15

**Authors:** Cinzia Perlini, Angelo Picardi, Alessandro Vinciarelli, Marcella Bellani

**Affiliations:** 1 Section of Clinical Psychology, Department of Neurosciences, Biomedicine and Movement Sciences, University of Verona, Verona, Italy; 2 Centre for Behavioural Sciences and Mental Health, Italian National Institute of Health, Rome, Italy; 3 School of Computing Science, University of Glasgow, Glasgow, United Kingdom; 4 Section of Psychiatry, Department of Neurosciences, Biomedicine and Movement Sciences, University of Verona, Verona, Italy

**Keywords:** attachment, attunement, healthcare, interpersonal physiology, psychotherapy, social cognition, therapeutic alliance

The concept of intersubjectivity, defined as the shared construction and perception of reality among individuals, has long attracted scholarly interest across multiple disciplines, serving as a conceptual bridge among mental health disciplines, philosophy, and, more recently, neuroscience (Bellani et al., 2026). Although a growing body of research has examined intersubjectivity in various dyadic relationships (e.g., mother–child, teacher–student interactions), a substantial gap remains in our understanding of intersubjective processes in clinical settings. In this specific context, both the patient and the clinician bring their individual motivations, affective states, behaviors, and relational patterns into the therapeutic encounter, conveyed through verbal and, especially, non-verbal cues. The shared relational space that emerges through this co-constructive process becomes a therapeutic element in itself, facilitating the restoration of psychological organization and integration in the patient (Lucherini Angeletti et al., 2025).

The clinical encounter, therefore, represents a unique relational context in which the interaction between clinician and patient plays a pivotal role in alleviating distress and fostering adaptive engagement with the social environment. Many scholars have underlined the epistemological significance of intersubjective experience, and disciplines as diverse as phenomenology, psychoanalysis, cognitive sciences, and neuroscience have acknowledged the relevance of one's own subjective perceptions to understanding the Other. Traditionally, key constructs within social cognition—including attachment, empathy, and therapeutic alliance—have been extensively investigated in psychological research. More recently, advances in social and affective neuroscience have introduced a novel perspective by emphasizing the physiological and neural mechanisms underlying intersubjectivity and offering new ways to observe relational phenomena. Despite these advances, important questions remain regarding how intersubjective processes emerge and operate within clinical contexts and how they may contribute to therapeutic or organizational effectiveness. This Research Topic aimed to advance knowledge by bringing together high-quality, innovative contributions investigating intersubjectivity in this field. Specifically, the included papers cover a variety of topics ranging from therapeutic relationship and alliance to clinician's attachment and attunement, interpersonal physiology, the diagnostic value of clinician's subjective experience during the interaction with the patient, and interpersonal dynamics in medical context. Overall, they adopt different methodologies (case report, clinical study, review, theoretical paper) across diverse settings (e.g., psychotherapy, hospital) and clinical conditions (e.g., psychotic disorders, depression, borderline personality disorder, spinal cord injury).

Concerning the therapeutic relationship and alliance, Brewer and Giommi argue that contemporary psychotherapy has become increasingly complex due to the proliferation of diagnostic categories, theoretical models, and intervention techniques. The authors propose curiosity, particularly interest-based curiosity, as a unifying and simplifying principle that can enhance therapeutic effectiveness by fostering psychological flexibility, openness, and a deeper patient-clinician alliance, while reducing rigidity and dependency within the therapeutic relationship. Curiosity is conceptualized not only as a therapist characteristic but as a trainable professional competence that supports insight, self-awareness, and therapeutic presence.

Moving from a theoretical to an applicative approach, Videtta et al. reviewed the literature on the role of therapeutic alliance in the treatment of Major Depressive Disorder (MDD). Seven out of ten studies selected by the authors indicated that a strong therapeutic alliance is associated with greater symptom reduction, improved functioning, enhanced treatment adherence, and lower risk of relapse. These findings were consistent across different psychotherapeutic approaches, clinical settings, and treatment delivery. By contrast, the remaining studies have suggested a reverse relationship, in which early symptom improvement strengthens patients' perceptions of the therapeutic alliance. Loreskär and Binder extend the investigation of the therapeutic alliance to developmental contexts, focusing on seriously ill and hospitalized children during art therapy. The study highlights how the therapeutic alliance is a central component of effective intervention. Through mirroring the child's language, offering choices, validating emotions, and allowing the child to maintain control over the artistic process, the therapist fostered a strong sense of emotional security, self-efficacy, and resilience. This supportive alliance enabled the children to reconnect with healthier aspects of themselves despite severe physical limitations, and helped them move from passivity and illness-focused experiences toward creativity, mastery, and a sense of personal achievement.

Høgenhaug, Kongerslev et al. and Høgenhaug, Steffensen et al. have explored in depth the physiological component of inter-subjectivity. Grounded in an embodied perspective, they first reviewed the relationship between alliance rupture–repair processes and interpersonal coordination dynamics in psychotherapy (Høgenhaug, Kongerslev et al.) and secondly applied this perspective to the treatment of borderline personality disorder, using a multimethod single-case design (Høgenhaug, Steffensen et al.). The review conceptualizes the therapeutic alliance as a dynamic process of mutual negotiation, continuously shaped by verbal and non-verbal interaction modalities and including movement synchrony, physiological arousal, vocal patterns, facial expressions, and linguistic matching. These coordination processes were interpreted as markers of mutual emotion regulation, empathic attunement, trust, and collaborative meaning-making, where rupture episodes were often characterized by disruptions in synchrony, whereas repair processes involved the restoration or transformation of coordinated interaction.

Fonzi et al. and Giannotti et al. address intersubjectivity by adopting the clinician as their primary analytical framework. The focus of Fonzi et al.'s work is intersubjectivity as it unfolds during the clinical encounter, particularly during diagnostic evaluation. Drawing on a phenomenological background, they re-examined the classical concept of *Praecox Feeling*, focusing on the clinician's subjective experience during encounters with patients affected by psychotic disorders. The study involved 49 clinicians and 326 patients and assessed clinicians' subjective reactions using the Assessment of Clinician's Subjective Experience (ACSE). They found that clinicians reported significantly greater Difficulty in Attunement when interacting with patients diagnosed with schizophrenia compared with those affected by delusional disorder or psychotic mood disorders. Despite some limitations, the findings support the notion that schizophrenia is characterized by a distinctive disturbance in intersubjective relatedness, perceived by clinicians as a sense of alienness or failure of emotional resonance. The study also provides empirical support for Rümke's hypothesis that subjective clinical impressions may contribute to diagnostic reasoning, as well as for the importance of the relational and intersubjective dimensions within psychiatric assessment. Giannotti et al. investigated psychotherapists' attachment organization and its relationship to therapists' ability to engage intersubjectively. They assessed psychotherapists, psychotherapy patients, and adults from the normative population using the Dynamic-Maturational Model Adult Attachment Interview (DMM-AAI). They found that 40% of psychotherapists exhibited high-risk attachment strategies similar to those seen in patients, while others demonstrated more adaptive profiles. Notably, psychotherapists were significantly more likely than patients to show evidence of “reorganization,” a process of conscious movement toward more balanced and integrated attachment functioning. The authors argue that therapists' personal attachment organization may influence their ability to establish intersubjectivity and therapeutic attunement with patients, with self-awareness and capacity to reflect on their own attachment-related vulnerabilities being more important for therapeutic effectiveness than specific treatment models. They conclude that psychotherapy training and supervision should place greater emphasis on therapists' attachment functioning, personal development, and relational competencies.

Extending the perspective on intersubjectivity to a clinical healthcare setting, Jiang et al. investigated self-perceived burden (SPB) among patients with spinal cord injury and examined its relationship to caregiver reactions. They reported that patients' perceptions of being a burden were significantly and positively associated with caregivers' emotional, physical, economic, and social responses, suggesting that patients' subjective experiences are shaped not only by physical disability but also by their perceptions of how others respond to their condition. Overall, the study supports a relational understanding of illness, in which psychological adjustment is co-constructed through reciprocal interactions between patients and significant others. The findings further emphasize the need for healthcare professionals to address both patient and caregiver experiences as interconnected aspects of the rehabilitation process.

Finally, Na et al., Ma et al., and Bai and Bai moved from a dyadic clinical setting to a group- and organizational-level, extending the analysis of relational processes beyond direct clinical encounters to the framework in which such encounters are embedded. Although their studies do not address clinical intersubjectivity in a strict sense, they provide insight into the broader relational conditions that may facilitate or constrain interpersonal openness and reciprocal exchange among healthcare professionals by shaping clinicians' engagement and professional growth. In particular, Na et al. analyzed questionnaires from 250 Chinese nursing trainees to investigate the role of incivility in professional training, conceived as a disrupted dimension of relationships in which rude speech, action, or behavior can cause psychological and physiological distress to the individuals involved. According to the study, trainees' incivility was strongly associated with weakened interns' sense of belonging, professional identity, and confidence, thereby increasing learning burnout, a phenomenon of particular concern worldwide. Career calling and self-efficacy partially mediated this relationship, indicating that internal psychological resources can buffer the effects of adverse interpersonal experiences. From an intersubjective perspective, the study suggests that the clinical learning environment is not merely a context for technical training but also a relational space in which recognition, respect, and supportive communication influence individual functioning. Similarly, the study by Ma et al. on 394 male nursing students in China suggested motivation/interest in the profession, self-consistency and congruence, psychological resilience, and perceived social/family support as principal predictors of a robust professional identity. Drawing on self-determination theory, the authors conceptualized relatedness as a fundamental psychological need and perceived social support as a positive experience arising from interactions with others. These findings suggest that professional identity is not shaped exclusively by individual dispositions but emerges within a relational field in which the self is continuously negotiated with significant others (educators, family members, and clinical mentors) and within the broader professional environment. Importantly, clinical experience itself appears to provide a relational context for this process. Finally, Bai and Bai used latent profile analysis in a sample of 523 clinical nurses, identifying three profiles: “Insiders,” “Go-betweens,” and “Outsiders,” based on nurses' differential perceptions of the organizational atmosphere. Nurses classified as “Outsiders” showed the highest levels of organizational silence, whereas “Insiders” showed the lowest, suggesting that perceptions of exclusion, unequal recognition, and relational distance can undermine trust, a sense of belonging, and willingness to express concerns or opinions within the healthcare team. The study highlights the importance of relational experiences in shaping professional communication and participation within clinical teams. Taken together, these latter contributions suggest that some of the relational processes underpinning dyadic intersubjective relationships—such as reciprocity, trust, recognition, respect, support, perceived relational climate, opportunities for self-expression, and interpersonal exchange—may also operate more broadly within the clinical context.

Collectively, the papers included in this Research Topic make a valuable contribution to the investigation of intersubjectivity in the clinical setting. Although not exhaustive, they may allow readers to better understand the complexity and multidimensionality of the relational dynamics that unfold when Self and Other meet in a context of care. Given the exponential technological advances in neuroscience in recent years, we anticipate that future research will further delineate the central and peripheral biological substrates underlying the key dimensions of intersubjectivity.
